# An evaluation of the effectiveness of a community mentoring service for socially isolated older people: a controlled trial

**DOI:** 10.1186/1471-2458-11-218

**Published:** 2011-04-08

**Authors:** Andy P Dickens, Suzanne H Richards, Annie Hawton, Rod S Taylor, Colin J Greaves, Colin Green, Rachel Edwards, John L Campbell

**Affiliations:** 1 Primary Care Research Group, Peninsula College of Medicine & Dentistry, University of Exeter, Smeall Building, St Luke's Campus, Magdalen Road, Exeter, EX1 2LU, United Kingdom; 2 Institute of Health Service Research, Peninsula College of Medicine & Dentistry, University of Exeter, Veysey Building, Salmon Pool Lane, Exeter, EX2 4SG, United Kingdom

**Keywords:** social isolation, complex intervention, controlled trial

## Abstract

**Background:**

Social isolation affects a significant proportion of older people and is associated with poor health outcomes. The current evidence base regarding the effectiveness of interventions targeting social isolation is poor, and the potential utility of mentoring for this purpose has not previously been rigorously evaluated. The purpose of this study was to examine the effectiveness of a community-based mentoring service for improving mental health, social engagement and physical health for socially isolated older people.

**Methods:**

This prospective controlled trial compared a sample of mentoring service clients (intervention group) with a matched control group recruited through general practice. One hundred and ninety five participants from each group were matched on mental wellbeing and social activity scores. Assessments were conducted at baseline and at six month follow-up. The primary outcome was the Short Form Health Survey v2 (SF-12) mental health component score (MCS). Secondary outcomes included the SF-12 physical health component score (PCS), EuroQol EQ-5D, Geriatric Depression Score (GDS-10), social activity, social support and morbidities.

**Results:**

We found no evidence that mentoring was beneficial across a wide range of participant outcomes measuring health status, social activity and depression. No statistically significant between-group differences were observed at follow-up in the primary outcome (p = 0.48) and in most secondary outcomes. Identifying suitable matched pairs of intervention and control group participants proved challenging.

**Conclusions:**

The results of this trial provide no substantial evidence supporting the use of community mentoring as an effective means of alleviating social isolation in older people. Further evidence is needed on the effectiveness of community-based interventions targeting social isolation. When using non-randomised designs, there are considerable challenges in the recruitment of suitable matches from a community sample.

**Trial registration:**

SCIE Research Register for Social Care 105923

## Background

In the United Kingdom, people aged 60 years or above currently account for approximately 20% of the population [1], and this proportion is expected to rise to 24% by 2030 [2]. By 2025 the number of people in the UK over the age of 80 is forecast to increase by almost a half, whilst those over 90 years will double [3]. This demographic shift has resulted in importance being placed on health status trends for older people and how these trends may change in future, due to the anticipated increase in demand for health and social care services [4]. More recently, longer life expectancy has led to discussion of the likely quality of life associated with these additional years [5,6]. 'Quality of ageing' is rapidly becoming one of the most important social, political and health priorities of the early 21st century. The development of strategies for promoting quality of ageing for older people has been a major component of recent UK Government policy [3,7-12]. Emphasis in many of these policies [7,10-12] was placed on 'active ageing', referring to enhancing quality of life by optimising older people's health, security and social participation in terms of learning, leisure, volunteering and employment.

As the proportion of older people in the population increases, more are living alone. A recent longitudinal UK study of people aged 50 or over reported that 22% (1363/6164) lived alone, of whom 13% experienced long periods of detachment from society [13]. Thus the challenge of addressing social isolation in this age group is a growing concern. A UK study [14] with a nationally representative sample of 999 people aged 65 years or over, found that between 11 and 17% of this age group reported being 'socially isolated'; defined as not being in direct contact with family, friends or neighbours on a weekly or monthly basis respectively.

A recent meta-analysis of 148 longitudinal studies (308,849 participants, mean age of 64 years) reported a 50 per cent reduction in the likelihood of mortality for individuals with strong social relationships [15]. A limitation of this review was that 'strong social relationships' was a composite variable that combined conceptually distinct measures of an individual's social context (e.g. loneliness, social isolation, perceived social support). Notwithstanding this, the authors observed that the impact of social relationships on mortality risk is comparable with major, well-established risk factors such as smoking and alcohol consumption, and exceeds that of physical inactivity and obesity. Studies focusing specifically on the measurement of social isolation and health report similar relationships. For example, nationally representative cross-sectional US survey data (n = 2910) reveal an association with poor self-rated physical health [16] and a three-year longitudinal study in Sweden (n = 1203) reported those who were socially isolated having increased susceptibility to dementia [17]. Both studies were based on the general population of older people, while a three-year Danish longitudinal study (n = 2,697) reported an association between social isolation and disability onset among older males that live alone [18]. In a separate analysis of data from our current study, we found that social isolation was negatively associated with health status and health-related quality of life of older people [19]. The wide-reaching implications of social isolation make targeting the problem a public health concern. In recent years, models of preventive joined-up local services have been developed to address social isolation and to promote wellbeing. Furthermore, one objective of current mental health policy [20] is to develop 'sustainable, connected communities' that promote social networks and environmental engagement.

In the UK, a model was devised ('Link Age') to offer older people one-stop access to services and advice through a single gateway. A 'Link Age Plus' service was subsequently introduced in pilot sites across the country in 2006, offering a fully integrated service with a specific focus on tackling social exclusion in older people [11]. However, little policy guidance or research evidence is available to define precisely how such services should be configured to optimise their effectiveness [21].

Cattan et al [22] conducted a systematic review to assess the effectiveness of interventions targeting social isolation and loneliness among older people. Thirty studies were included, targeting a variety of groups such as caregivers, those living alone and general populations of community-dwelling older people. The authors judged nine studies to be of high methodological quality, four studies to be high/moderate quality, with the remaining 17 studies being of lower quality. The authors characterise interventions that effectively alleviate social isolation as being those delivered at the group level, offering education or social activity and targeting specific groups of older people. One-to-one interventions such as home visiting or befriending were reported to be not effective [22,23]. The latter finding has been supported by two more recent studies. Charlesworth et al [24] conducted a randomised controlled trial (RCT) examining the effectiveness of befriending for carers of people with dementia (n = 236). The authors reported no intervention effect on mood or health-related quality of life at 15 months. A systematic review of eight RCTs evaluating home visiting programmes for older people with poor health [25] reported no beneficial effects on mortality or health status. Despite the above generic characteristics of effective services, Cattan et al [22] and Findlay [21] acknowledge that there remain many more questions than answers, due in part to the poor quality of the evidence base limiting interpretation of results.

An alternative intervention proposed for tackling social isolation is mentoring. The purpose of a mentor is to provide support within a given context, with the level of support offered being variable and responsive to the perceived needs of the recipient [26]. Mentoring has been defined as a 'unique learning partnership' offering either emotional or instrumental support [27].

### Early Mentoring service development and evaluation

Healthy Living Centres (HLC) were introduced to local communities in 1999 with the aim of reducing health inequalities [28]. The Upstream HLC was specifically designed to increase social participation of older people at risk of social isolation living in a rural county of South West England, through the provision of a mentoring service. The intervention involved training mentors to facilitate older people's participation in individually-tailored creative and social activities with mentors reducing the level of support over time as appropriate. The aim of the intervention was to restore older people's self-confidence, self-esteem and social identity, and to support their participation and re-engagement in community-based activities.

An observational study of this intervention assessed clients at baseline, six months and 12 months post intervention [29]. Baseline scores indicated poor mental and physical health compared with age-matched population norms and low levels of social support. At six months, there were significant improvements in self-reported mental wellbeing (SF-12 mental health component score [30]) and depression scores, but not in physical health or social support. At 12 months, the improvement in SF-12 mental health component scores was not maintained although there were significant improvements in mental health status (depression) and social support and a trend towards improvement in SF-12 physical health component scores [30] (p = 0.06).

Qualitative data showed that the intervention was well-received by participants. Key mediators of beneficial outcomes appeared to be the individual tailoring of the intervention to clients' needs and overcoming barriers relating to confidence, transport and venues. Key processes underlying improved outcomes were the development of a positive group identity, and the building of confidence and self-efficacy. The model appeared to provide a practical way of engaging socially isolated older people and generating social networks. Although there are limitations in attributing causality in uncontrolled observational studies, the data indicated that the use of mentoring for social isolated older people merited further investigation.

On the basis of findings from the pilot study, funding was secured to sustain and develop the mentoring service and to expand the model across the county of Devon by commissioning new providers to implement a 'Devon Community Mentoring service'.

Devon has a population of approximately 1.1 million, with a high proportion of older people compared with the UK. Forty one percent are 50 years of age or above and 20% are at least 65, in comparison with 34% and 16% of the UK population respectively [31]. Devon is a largely rural county with a population density of less than a third that of England, and half that of the UK [32].

### Description of the Devon Community Mentoring Model

The underlying principles and definitions guiding the implementation and operationalisation of the Community Mentoring service have been documented in a manual [33]. Community Mentoring was delivered by two main voluntary organisations, through operational clusters across Devon. Mentoring teams worked to identify older people (aged 50 or over) who were, or were at risk of becoming socially isolated and who, they believed, might benefit from mentoring. The intervention was intended for those with substantial psychological or physiological morbidity, depression, chronic illness, disability, poor quality of life or substantial caring burden. Exclusion criteria included a history of violence, alcohol dependency, psychosis or more than mild dementia. Potentially eligible clients were identified via referrals from health and social care professionals, family and friends, or self-referral. Individuals were assigned a mentor who worked with them for up to 12 weeks; in rare cases the support could be offered for longer. By working closely with clients, mentors aimed to build clients' self-confidence and engage them in personally meaningful social activities. A key goal was to provide clients with the necessary skills and abilities to ensure sustainable change once the service was withdrawn.

### Purpose of the study

The aim of the study was to examine the effectiveness of the community-based mentoring intervention compared with usual care, in improving mental health, social engagement and physical health.

## Methods

### Study design and ethical considerations

A prospective controlled trial was conducted. Potential participants were identified from a population of individuals who were currently in receipt of mentoring (intervention), or who were receiving usual care through routinely available health, social and voluntary care services (control). Intervention and control group participants were matched on two variables associated with the anticipated effect of the intervention (mental wellbeing and social activity) to minimise potential confounding effects.

The study sample size was calculated to detect a significant between-group difference on the primary outcome measure (SF-12 MCS). The previous observational study [34] reported a change in SF-12 MCS from baseline to follow-up of 3.8 points (95% CI: 0.89 to 6.68, p < 0.02). An improvement of ≥3 points on the SF-12 summary scores was deemed to be clinically meaningful [35]. Were this effect to be replicated and assuming that the between-group difference would be three points or more in this study, a minimum of 140 participants per group were required (two-sided alpha = 0.05, 85% power). To account for likely loss to follow-up in this age group [36,37] an additional 25% was needed, resulting in a target sample size of 187 participants per group.

Formal approval for this study was granted by Devon & Torbay NHS Research Ethics Committee (REC number 07/Q2102/9) and the appropriate NHS trusts and social care providers across Devon.

### Participant recruitment and outcome measures

Participant recruitment took place between March 2007 and June 2008.

Intervention and control group participants were required to meet the study inclusion criteria of being 50 years of age or above, being socially isolated or at risk of becoming socially isolated, being able to provide informed consent, and being able to complete a questionnaire with or without assistance. Potential participants were excluded from the study if they had dementia, psychosis or alcohol dependency, or lived in a nursing home.

Community Mentoring teams were requested to invite all new clients to participate in the study. Mentors were asked to mention the study and offer clients a study information pack. The pack consisted of a brief study flyer, a participant information sheet, an invitation from the research team and a response form. Interested clients were invited to return the response form directly to the research team using a pre-paid envelope. On receipt of completed response forms, interested clients were contacted to arrange a home visit to discuss the study further before providing consent and completing the baseline questionnaire. Intervention participant baseline assessments were conducted as quickly as possible after the mentoring team's initial assessment, but before the mentor began actively working with the client.

Potential control participants were sampled from areas of Devon where the mentoring service was not currently available. The sample was recruited through three general practices covering areas with similar population demographics to the mentoring service catchment areas. Surgery staff identified the patients on the practice lists that were aged 50 or above. Clinical staff then excluded patients who did not meet the study inclusion criteria or who were diagnosed with a terminal illness or were classed as temporary residents. The latter exclusions were for ethical and pragmatic reasons, as screening surveys were to be sent out un-invited, requesting people's involvement over a considerable period of time.

Study information and screening surveys were sent to a random sample (approx 20%) of the remaining patients who were eligible for the control group. The brief screening survey included participant socio-demographic information and a selection of outcome measures (SF-12, social activity, social support) that were used in the study assessments. Since the aim of the trial was to examine the effectiveness of the mentoring intervention, only those outcome measures required for matching purposes or for describing our samples were included in the screening survey. The rationale for the selection of matching criteria is described subsequently. Non-respondents were sent a second screening survey and accompanying material approximately two weeks later. All recipients were given the option to complete the survey only, or to complete the survey and express an interest in taking part in the study as a control group participant. If the respondent was willing to take part and they were selected as a suitable match with an intervention participant, the researcher telephoned the person to discuss the study further and to arrange a home visit for consent and baseline data collection.

Matching was performed on a case-by-case basis, so that each intervention group participant was matched with one person from the community sample who returned the screening survey and was willing to participate in the trial. Data used to match pairs were obtained from intervention participants' baseline assessments and from control participants' completed screening surveys. Matching was therefore an ongoing process throughout the recruitment phase of the trial. Selection of matching variables was informed by the anticipated effects of the intervention as well as the observed improvement in MCS (derived from the SF-12) in the earlier observational study. Pairs were matched using mental health status and social activity scores, both reflecting one of five categories. Using the mean and standard deviation (SD) for the sample of intervention group participants available (n = 49), the five categories were: 'low outlier' (> -2 SD from mean), 'poor' (> -1 SD from mean), 'medium' (within ±1 SD), 'good' (> +1 SD from mean) and 'high outlier' (> +2 SD from mean). Pairs of participants were matched to be in the same category of mental health status and social activity scores. Where this was not possible, pairs were matched on one criterion and within one category shift on the other. The described matching process therefore intended to ensure that matched pairs had comparable levels of 'baseline risk' [38] in respect of mental health and social activity.

There was a trade-off between the number of matching criteria applied and the ability to identify suitable controls within the time frame. The two matching criteria used in this study both had five categories, meaning that all participants were allocated to one of 25 categories (5 × 5) prior to matching. An additional matching category might have been desirable (e.g. gender or marital status [widowed/not widowed]), and could potentially have reduced the disparity between groups observed at baseline. However, the availability of suitable matches (participants would have been allocated into 5 × 5 × 2 = 50 groups) may have diminished to a point where the trial was not achievable in the time frame available [39].

Baseline assessments for matched pairs were conducted within as short a time period as possible, up to a maximum of three months apart, to minimise the possible impact of seasonal variation on mental health [40]. Study participants completed a follow-up assessment six months after baseline data collection. This length of follow-up was chosen to capture any benefits maintained after mentoring services had been withdrawn.

The measures taken at baseline and follow up were selected to reflect areas where the intervention might be expected to impact on health and well-being. We selected instruments that have been widely used in previous research, or that were seen to be both acceptable to the population group and sensitive to change in the previous observational study [29]. The primary outcome was mental health status, as measured by the SF-12 mental health component score (MCS) [41]. Secondary outcome measures included the SF-12 physical health component score (PCS), health status (EuroQol EQ-5D) [42], Geriatric Depression Scale (GDS-10) [43], and social health including social activities (four items from the RAND Social Health Battery [44]), social support (six items from the Medical Outcomes Study Social Support Survey [MOS-SSS] [45]), and levels of social participation (one item from the General Household Survey).

The social support items used were selected and validated during the preceding observational study, following reports from participants that the 19-item Social Support Survey was repetitive and tedious [29]. The authors derived a single scale (referred to as MOS-6) from these items, with scores ranging from one to five, where higher scores reflected more positive assessments of the functional support available. The items were found to have good internal consistency (α = 0.93), and formed a single factor with loadings ≥0.76. The MOS-6 explained 81% of the variance in the 19-item MOS-SSS. Furthermore, the MOS-6 demonstrated criterion validity, being sensitive to change in the context of a social isolation intervention [29] and having moderate correlations with both the SF-12 MCS (r = 0.41) and the GDS (r = 0.48).

### Analysis plan & reporting

The study was reported according to the STROBE statement, specifying the minimum required information for good quality reporting of observational studies [46].

Data were analysed on an intention to treat basis. Pre-specified between-group comparisons at 6-months follow up were undertaken using regression analyses. In spite of the matching process used, given the lack of comparability of groups at baseline (see Results) an unpaired approach to analysis was undertaken. Statistical models included adjustment for baseline scores and for employment status, living arrangements and accommodation type. Further statistical models including the additional covariates of age and gender were also run, though results from these models are not reported as the additional adjustment did not change the between-group inferences (data not presented).

Whether or not P-values should be adjusted for multiple comparisons is controversial [47]. We elected not to adjust for multiple comparisons because of the increased likelihood of Type II errors. We considered all statistical tests as significant at a two-sided P of less than 0.05 and results are expressed as 95% confidence intervals.

The main reason for missing data was anticipated to be loss to follow up. Missing values were imputed using last observation carried forward [48], assuming that missing data were randomly distributed. The results of regression modelling, with and without imputation, were compared to assess the potential impact of using this method.

All statistical analyses were conducted using STATA version 10.

## Results

### Recruitment

**Figure 1:**
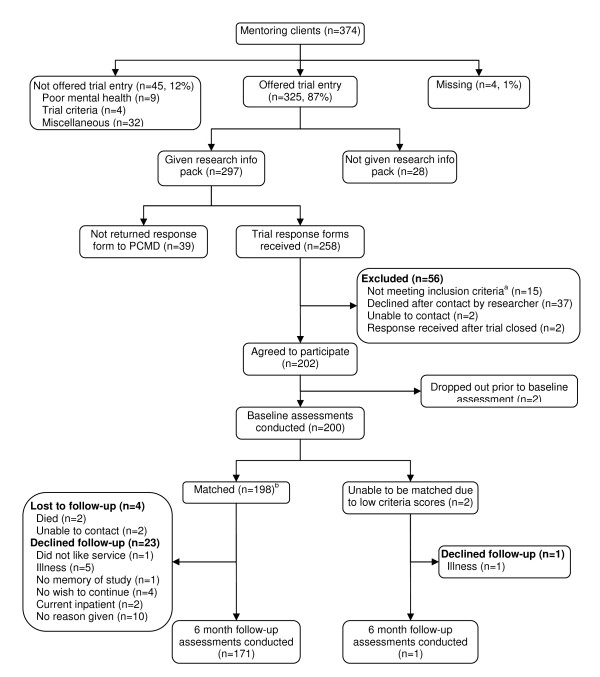
**Intervention group recruitment flow chart**. ^a ^Reasons included: not receiving mentoring (n = 4), received signposting only (n = 3), current inpatient (n = 2), poor mental health (n = 2), not new mentoring client (n = 2), nursing home resident (n = 1), did not want to meet researcher (n = 1) ^b ^This figure equates to the 195 'matched' controls in Figure 2. Three intervention participants declined follow-up resulting in their matched controls being reassigned to another intervention group participant.

Of the 765 individuals referred to mentoring teams during the recruitment period, the research team was able to identify 374 people who were eligible for mentoring, had been assessed by a mentor and who subsequently received the service. Of these, 87% (325/374) were offered trial entry by a mentor. Of the mentoring clients offered trial entry, 200/325 (62%) consented to participate (Figure 1).

There were 16070 potentially eligible patients on the lists of the three control practices. Researchers identified a random sample of patients (n = 3700) that was then screened by practice staff for exclusions (n = 154), resulting in the final patient sample approached (n = 3546). A total of 2057 responses were received, of whom 901 (25.4% of those approached) were interested in participating in the trial. The interested community respondents appeared to have a similar profile to the broader random sample of older people approached through practice lists in terms of their age, gender and IMD deprivation scores (data not presented).

**Figure 2:**
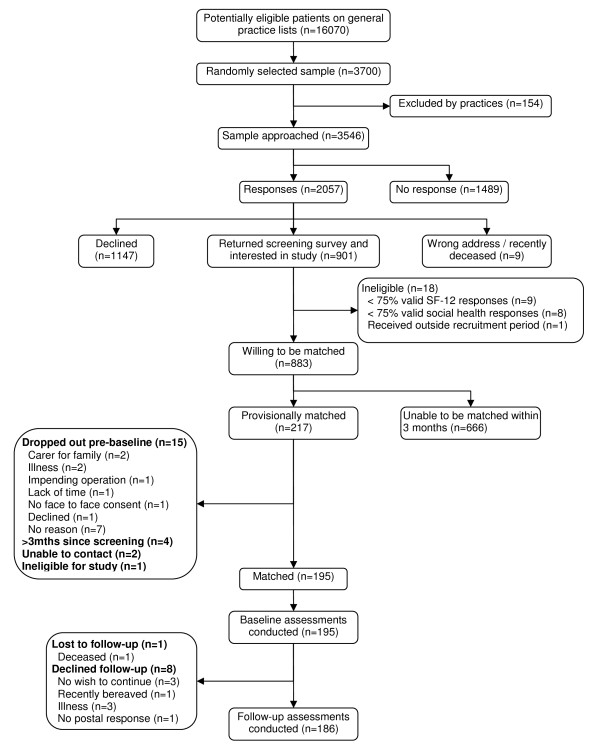
**Control group recruitment flow chart**.

Two thirds (138/200, 69%) of intervention group participants providing baseline data had a score of 'moderate' or worse on both SF-12 MCS and social activity matching criteria. In comparison, only one third (360/884, 41%) of the interested community respondents who provided sufficient data had scores of 'moderate' or worse. An implication of this was that a considerable number of screening survey respondents would not be able to be matched as their profiles corresponded with only a small proportion of intervention participants with higher scores. Of the interested screening survey respondents, 195 individuals were matched to intervention group participants based on their screening survey data (Figure 2).

### Representativeness of participants

The recruitment target of 187 participants per group was achieved. Although we encouraged mentoring teams to offer their clients entry to the trial, we found evidence of selection bias. Community Mentoring clients who were not offered study entry by mentors were from more deprived backgrounds and reported significantly lower levels of social activity than those offered trial entry. There was also evidence of recruitment bias, as clients who were offered trial entry but who declined to take part were also significantly older, were less socially active than individuals who agreed to take part, and were from more deprived backgrounds (p < 0.05 in all cases, data not presented).

### Adequacy of matching

A cross-tabulation of matching criteria scores for control and intervention groups at the point of matching (control group screening survey and intervention group baseline assessments) demonstrated that the groups were comparable on both matching criteria (MCS: z = -1.22, p = 0.22; social activity: z = -1.28, p = 0.20). However, when this analysis was repeated on baseline data for both groups, the intervention group reported lower scores on both SF-12 MCS (z = -5.76, p < 0.001) and social activity scores (z = -4.12, p < 0.001) compared with controls. The average time (SD) between receipt of a completed screening survey and a control participant's baseline assessment was 61.9 (24.8) days. The majority of control group participants completed baseline assessments within three months of returning their screening surveys, with only six (3.1%) participants being assessed outside this period.

### Characteristics of participants

A summary of the socio-demographic characteristics and living circumstances of trial participants is presented in Table 1. There were differences in the profiles of participant groups at baseline. Intervention group participants were less likely to be married or living as married and more likely to be living alone. In addition, fewer intervention group participants were home owners or in active employment in comparison with the control group. The groups were however comparable in respect of age, gender and ethnicity.

**Table 1:** Baseline characteristics of trial participants

	Intervention participants (n = 200)	Control participants (n = 195)	**Test statistic**; p-value
Age - mean (SD)	71.8 (12.2)	69.8 (11.6)	-1.7; p = 0.1^a^

Gender - n (% male)	62 (31.0)	76 (39.0)	2.8; p = 0.1^b^

Ethnicity - n (%)			4.7; p = 0.2^b^
White British	172 (86.0)	180 (92.3)	
White European	7 (3.5)	3 (1.5)	
Other ethnic group	20 (10.0)	12 (6.2)	
Ethnicity not disclosed	1 (0.5)	-	

Lives alone - n (%)			36.2; p < 0.001^b^
Yes	124 (62.0)	62 (31.8)	
No	76 (38.0)	133 (68.2)	

Marital status - n (%)			42.7; p < 0.001^b^
Single, never married	18 (9.0)	7 (3.6)	
Separated or divorced	34 (17.0)	20 (10.3)	
Widowed	87 (43.5)	45 (23.1)	
Married, or living as married	61 (30.5)	123 (63.1)	

Accommodation type - n (%)			72.6; p < 0.001^b^
Home owner	113 (56.5)	181 (92.8)	
Rented/Council	82 (41.0)	14 (7.1)	
Nursing home	1 (0.5)	-	
Residential home	4 (2.0)	-	

Employment status - n (%)			56.9; p < 0.001^b^
Employed	5 (2.5)	53 (27.2)	
Unemployed	7 (3.5)	2 (1.0)	
Long term sick or disabled	33 (16.5)	10 (5.1)	
Retired	155 (77.5)	130 (66.7)	

^a ^Two-sample t test

^b ^Chi-square test

### Baseline outcome scores for participants

Differences were observed between intervention and control group participants at baseline (Table 2). In general, the control group had significantly better levels of mental, physical and social health compared with intervention participants.

**Table 2:** Baseline outcome scores

Health measures	n	Intervention participants	n	Control participants	**Test statistic**; p-value
SF-12 MCS - mean (SD)	197	43.2 (11.0)	195	49.6 (9.8)	6.1; p < 0.001^a^

SF-12 PCS - mean (SD)	197	34.7 (11.7)	195	42.8 (12.5)	6.6; p < 0.001^a^

EQ-5D - mean (SD)	198	0.5 (0.3)	194	0.7 (0.3)	7.5; p < 0.001^a^

GDS-10 - mean (SD)	195	4.4 (2.4)	192	2.2 (2.2)	-9.3; p < 0.001^a^

**Social support**	**n**		**n**		**Test statistic**; **p-value**
MOS-6 - mean (SD)	199	2.7 (1.1)	193	3.5 (1.0)	8.3; p < 0.001^a^

**Social activities**	**n**		**n**		**Test statistic**; **p-value**

No. friends/family - median (IQR)	200	5 (2, 10)	193	5 (3, 10)	0.9; p = 0.38^a^

No. clubs/groups - median (IQR)	199	1 (0, 2)	194	1 (0, 2)	1.4; p = 0.15^a^

Get together with friends/family - n (%)	199		194		-0.9; p = 0.36^b^
Every day		14 (7.0%)		16 (8.3%)	
Several days a week		51 (25.6%)		50 (25.8%)	
Once a week		52 (26.1%)		49 (25.3%)	
2-3 times a month		24 (12.1%)		30 (15.5%)	
Once a month		16 (8.0%)		24 (12.4%)	
5-10 times a year		11 (5.5%)		14 (7.2%)	
Less than 5 times a year		31 (15.6%)		11 (5.7%)	

Getting along with others - n (%)	200		194		-1.6; p = 0.11^b^
Better		17 (8.5%)		16 (8.3%)	
The same		154 (77.0%)		164 (84.5%)	
Worse		29 (14.5%)		14 (7.2%)	

**GHS items**	**n**		**n**		**Test statistic**; **p-value**

Work around the house - n (%)	200	16 (8.0%)	195	36 (18.5%)	9.5; p < 0.01^c^

Transport/errands - n (%)	200	27 (13.5%)	195	59 (30.3%)	16.3; p < 0.001^c^

Child care - n (%)	200	21 (10.5%)	195	37 (19.0%)	5.7; p < 0.05^c^

Practical advice - n (%)	200	24 (12.0%)	195	37 (19.0%)	3.7; p = 0.06^c^

Emotional support - n (%)	200	48 (24.0%)	195	103 (52.8%)	34.7; p < 0.001^c^

Other - n (%)	200	26 (13.0%)	195	38 (19.5%)	3.1; p = 0.08^c^

None of the above - n (%)	200	107 (53.5%)	195	55 (28.2%)	26.1; p < 0.001^c^

**Morbidity**	**n**		**n**		**Test statistic**; **p-value**

Angina - n (%)	200	30 (15.0%)	195	14 (7.2%)	6.1; p < 0.05^c^

Arthritis - n (%)	200	92 (46.0%)	195	90 (46.2%)	0.001; p = 1.0^c^

Cancer - n (%)	200	15 (7.5%)	195	10 (5.1%)	0.9; p = 0.33^c^

Diabetes - n (%)	200	28 (14.0%)	195	17 (8.7%)	2.7; p = 0.10^c^

Heart failure - n (%)	200	17 (8.5%)	194	4 (2.1%)	8.1; p < 0.01^c^

High blood pressure - n (%)	199	98 (49.3%)	195	78 (40.0%)	3.4; p = 0.07^c^

Sight/hearing problems - n (%)	200	114 (57.0%)	195	73 (37.4%)	15.2; p < 0.001^c^

Stroke - n (%)	199	13 (6.5%)	195	2 (1.0%)	8.2; p < 0.01^c^

Depression or anxiety - n (%)	200	127 (63.5%)	195	66 (33.9%)	34.7; p < 0.001^c^

Memory/concentration problems - n (%)	200	121 (60.5%)	195	79 (40.5%)	15.8; p < 0.001^c^

Chronic respiratory conditions - n (%)	200	39 (19.5%)	195	30 (15.4%)	1.2; p = 0.28^c^

Sleeping difficulties - n (%)	200	120 (60.0%)	195	87 (44.6%)	9.4; p < 0.01^c^

^a ^Two-sample t test

^b ^Mann-Whitney U test

^c ^Chi-square test

Due to the imbalance in the characteristics and baseline outcome scores between intervention and control group participants, the modelling framework for our primary and secondary analysis strategy was altered. Covariates were included reflecting key differences in employment status (employed versus not), living arrangements (living alone versus not), accommodation type (home owner versus not), as well as the baseline score for the outcome of interest.

### Six month follow-up data

Most study participants who completed a baseline interview also completed a follow-up interview, with only 9% (37/395) dropping out across the six month period.

There was no evidence of a significant between-group difference in the primary outcome of SF-12 MCS (mean difference 0.8; 95% CI -1.5 to 3.2; p = 0.48) (Table 3). No significant between-group differences were observed for most of the secondary outcome measures with the exception of health status (EQ-5D) and one social activity item ('getting along with others'). Intervention group participants reported significantly less improvement in EQ-5D at follow-up than controls (mean difference -0.1; 95% CI -0.1 to -0.03; p < 0.01) and the degree to which they were getting on with other people (odds ratio 0.6; 95% CI 0.4 to 0.9; p < 0.01) had deteriorated compared with control participants.

**Table 3:** Between-group regression model comparing outcomes at six months

Health measures	Intervention 6-months	Control 6-months	Between-group difference^a, b^
SF-12 MCS - mean (SD)	n = 168 46.7 (11.2)	n = 182 49.2 (10.0)	0.8 (-1.5, 3.2); p = 0.48

SF-12 PCS - mean (SD)	n = 168 34.8 (11.4)	n = 182 42.7 (12.6)	0.1 (-1.9, 2.1); p = 0.90

EQ-5D - mean (SD)^c^	n = 172 0.6 (0.3)	n = 186 0.8 (0.2)	-0.09 (-0.14, -0.03); p < 0.01

GDS-10 - mean (SD)	n = 165 4.1 (2.4)	n = 185 2.2 (2.1)	0.2 (-0.2, 0.7); p = 0.29

**Social Activities**			

Nos. friends/family - median (IQR)	n = 172 5 (3, 8)	n = 183 6 (4, 10)	0.1 (-1.4, 1.6); p = 0.89

Nos. clubs/groups - median (IQR)	n = 172 1 (0, 2)	n = 186 1 (0, 2)	0.3 (-0.1, 0.6); p = 0.15

Get together with friends/family- median (IQR)^d^	n = 172 3 (2, 5)	n = 186 3 (2, 4)	1.5 (0.7, 3.2); p = 0.25

Getting along with others- median (IQR)^e^	n = 172 2 (2, 2)	n = 186 2 (2, 2)	0.6 (0.4, 0.9); p < 0.01

**Social support**			

MOS-6 - mean (SD)	n = 170 2.9 (1.1)	n = 184 3.6 (1.0)	0.03 (-0.2, 0.2); p = 0.75

**GHS items**			

Work around the house- n (%)	n = 171 10 (5.9%)	n = 186 32 (17.2%)	1.2 (0.5, 2.9), p = 0.72

Transport/errands - n (%)	n = 171 20 (11.7%)	n = 186 57 (30.7%)	1.5 (0.8, 3.0); p = 0.24

Child care - n (%)	n = 171 20 (11.7%)	n = 186 35 (18.8%)	1.2 (0.4, 3.5); p = 0.79

Practical advice - n (%)	n = 171 19 (11.1%)	n = 186 34 (18.3%)	1.1 (0.5, 2.5); p = 0.79

Emotional support - n (%)	n = 171 46 (26.9%)	n = 186 83 (44.6%)	1.2 (0.7, 2.2); p = 0.46

Other - n (%)	n = 171 29 (17.0%)	n = 186 42 (22.6%)	1.1 (0.6, 2.2); p = 0.76

None of the above - n (%)	n = 171 85 (49.7%)	n = 186 51 (27.4%)	0.7 (0.4, 1.2); p = 0.19

**Morbidity**			

Depression or anxiety - n (%)	n = 172 96 (55.8%)	n = 185 67 (36.2%)	1.0 (0.6, 1.9); p = 0.92

Memory/concentration problems- n (%)	n = 171 91 (53.2%)	n = 186 71 (38.2%)	0.9 (0.5, 1.7); p = 0.69

Sleeping difficulties - n (%)	n = 172 89 (51.7%)	n = 186 87 (46.8%)	1.2 (0.6, 2.3); p = 0.61

^a ^Adjusted for employment status, accommodation type and living circumstances at baseline.

^b ^Between-group differences for SF-12 MCS, SF-12 PCS, EQ-5D, GDS-10, no. of close friends/family, no. of clubs/groups and MOS-6 are presented as mean differences (intervention minus control), with odds ratios (control as reference category) presented for all other items to which intervention group is compared.

^c ^Presented to two decimal places as 95% CIs were unclear when using one decimal place due to rounding.

^d ^Scale: 1 = every day, 2 = several times a week, 3 = about once a week, 4 = 2 or 3 times a week, 5 = about once a month, 6 = 5 to 10 times a year, 7 = less than 5 times a year. An odds ratio >1 reflects the intervention group reporting poorer performance relative to the control group.

^e ^Scale: 1 = better than usual, 2 = about the same as usual, 3 = not as well as usual. An odds ratio >1 reflects the intervention group reporting better performance relative to the control group.

The effect of imputation was examined, with no qualitative differences observed between the imputed and non-imputed regression models except for one social activity item ('getting along with others'). While a significant between-group difference was reported for 'getting along with others' in Table 3 the imputed analysis produced a marginally non-significant difference (odds ratio 0.6; 95% CI 0.4 to 1.0; p = 0.06). Data from the non-imputed analyses, adjusted for the three covariates of employment status, living arrangements and accommodation were therefore reported for the purposes of interpreting the trial results.

## Discussion

### Summary of findings

At follow-up there was no evidence of a between-group difference for the primary outcome measure (SF-12 MCS) and for the majority of secondary outcome measures. These data suggest that there were no robust improvements in the health and wellbeing of individuals using the Community Mentoring service compared with controls at six months.

### Added value

This study was the first controlled trial of a mentoring service for community-dwelling socially isolated older adults. While there is no comparable effectiveness data regarding the use of mentoring with socially isolated older people, the between-group trial data did not reflect improvements in mental health status and in depressive symptoms that were reported in the earlier observational study [29]. It should be noted that data from the previous study represented within-group changes due to the uncontrolled design. While the within-group change in SF-12 MCS in our controlled trial was comparable with that observed in the previous study (3.5 vs 3.8), this should be interpreted cautiously due to substantive methodological differences in data collection and attrition. In the observational study, mentors collected data as opposed to independent researchers, and there was a 47% loss to follow-up as opposed to 9% in the controlled trial.

Our findings contrast with studies evaluating the effect of mentoring on health outcomes in other contexts, although the differences in populations studied should be noted. For example, a randomised controlled trial reported that patients aged 60 or above who were diagnosed with ischaemic heart disease demonstrated improved physical activity following mentor-led monthly meetings over a one-year period [49]. An uncontrolled, before and after study found that overweight adults with developmental disabilities reported improved lifestyle and weight loss following participation in a peer mentor-led health promotion programme over a seven month period [50]. The intervention consisted of twice-weekly education and exercise sessions, focusing on health, nutrition and fitness. A qualitative study suggested that mentoring adults with prior artistic interests in the uptake of creative activities promoted psychological wellbeing [51].

This trial has contributed to methodological considerations in the use of matched trial designs to evaluate a social intervention. Deterioration in the comparability of groups on matching criteria scores between the point of matching and the baseline assessment demonstrated the difficulty of matching participants using different time points, based on data collected via different modes of administration. Using baseline scores from both groups to match participants would have improved comparability, though this would have required substantial over-sampling of the potential control group to identify a subgroup whose baseline scores closely matched those of the intervention group.

### Strengths and limitations

In addition to its controlled design, this study had two particular strengths. Firstly, participant recruitment exceeded the sample size target (n = 187 per group, with 85% power) required to demonstrate potentially important between-group differences on the primary outcome measure (SF-12 mental health component score). Secondly, participant retention was also better than anticipated, with only nine percent of participants being lost to follow up. Hence, we feel confident that the follow-up data are unlikely to be substantially affected by attrition bias [52].

However, the study also has potential limitations. Firstly, the selection and recruitment bias in the intervention group suggested that mentoring clients contributing to the study may not have been representative of the broader pool of mentoring clients from which they were recruited. The trial findings therefore may not be generalisable to more socially isolated older people.

Secondly, the controlled trial design is inherently vulnerable to bias when compared with a randomised controlled trial. While randomised designs are considered to be methodologically superior when assessing questions of effectiveness [53], ethical and pragmatic challenges precluded their use in this study. The trial adopted various recruitment and matching procedures in an attempt to prevent or limit the potential for major imbalances between groups [46]. Despite this, important imbalances were evident at baseline that required accounting for in the primary analysis. The potential for misleading estimates of effect due to the limitations of the trial design cannot be conclusively ruled out and the findings should therefore be interpreted with caution.

It could be argued that the choice of matching criteria limited the comparability of study participants, as additional or alternative criteria could have been used. The selection of matching criteria necessarily balanced theoretical and methodological considerations against pragmatic issues. While attempting to match participants on more than two criteria may have resulted in more comparable controls, it would have become increasingly difficult to match sufficient numbers of participants within the trial timeframe.

### Reflections on trial results

Alternative explanations as to why no effect of mentoring was found in this study could relate either to methodological issues described previously, to intervention fidelity arising through variations in service implementation or to the targeting of clients. Although providers were commissioned to deliver the model specified within the Mentoring Manual, there was potential for considerable variation in the way that the service was implemented across operational clusters [54,55]. For example, there could be a lack of consistency in the assessment procedures between providers, in the competency and intervention delivery skills of mentors or in the types of activities offered to service clients. In addition, judgement regarding what constituted a sufficient 'dose' of mentoring could vary between providers. Older people (aged 50 or above) were considered eligible for the intervention if they were, or were at risk of becoming socially isolated and might benefit from the support of a mentor. Such broad eligibility criteria could result in a heterogeneous client group, with some clients unlikely to benefit from the intervention who may have been deemed ineligible under tighter criteria. Such a sub-group of clients could mask any positive gains in outcomes observed in the clients for whom mentoring was best suited.

Analysis of descriptive data collected during participant recruitment into the trial found that mentoring clients appeared to be different from the sample of community residents responding to the screening survey. Mentoring clients were more likely to be living alone and to report lower levels of social activities; suggesting that mentoring providers were targeting a potentially 'at risk' group within the community. Additionally, their mental and physical health status (SF-12) scores were substantially lower than UK normative data for similar age groups [56].

## Conclusions

The results of this trial provide no substantial evidence supporting the use of community mentoring as an effective means of alleviating social isolation in older people. While the Community Mentoring service did include some components of effective interventions (participatory, group-based) identified in previous literature [21,22], other components may not have been sufficiently addressed. Further work is required to develop and more rigorously evaluate interventions that target specific groups of socially isolated older people, deliver high quality training of facilitators and involve older people in intervention development.

There is a need for service providers to clearly define the characteristics of interventions, both in terms of their content and their target population, and to ensure intervention fidelity. Researchers will then be able to better specify details of the intervention being studied; improving the ability to interpret findings through process analyses [57], and to determine their generalisability to other populations and their implications for wider public health. In combination with the adoption of high quality, preferably randomised trial designs, such research would provide a valuable contribution to the evidence base on the effectiveness of complex, community-based interventions for socially isolated older people.

## Competing interests

The authors declare that they have no competing interests.

## Authors' contributions

APD was responsible for trial management, co-authored the protocol and ethical submission, had input into the data analyses, contributed to the interpretation of study data and wrote the paper. SHR was Principle Investigator for the study, co-authored the protocol and ethical submission, had input into the data analyses, and contributed to the interpretation of study data and to revisions to the paper. AH co-authored the protocol, and contributed to the interpretation of study data and to revisions to the paper. RST co-authored the protocol, performed the statistical analyses and contributed to the interpretation of study data and to revisions to the paper. CJG co-authored the protocol, contributed to the interpretation of study data and to revisions to the paper. CG contributed to the interpretation of study data and to revisions to the paper. RE contributed to revisions to the paper. JLC contributed to the interpretation of study data and to revisions to the paper. All authors have read and approved the final version of this manuscript.

## Pre-publication history

The pre-publication history for this paper can be accessed here:

http://www.biomedcentral.com/1471-2458/11/218/prepub
